# Supplementary material for: The impact of remote home monitoring of people with COVID-19 using pulse oximetry: A national population and observational study
Source: eClinicalMedicine. 2022 Mar 1;45:101318. doi: 10.1016/j.eclinm.2022.101318 (PMC8886180; doi:10.1016/j.eclinm.2022.101318)
Supplement: Supplementary file 1 [file mmc1.pdf]

The impact of remote home monitoring of people with COVID-19 using pulse oximetry: a national population and observational study.

Supplementary material

Contents

1. Data sources page 2
2. Judging completeness of data within CCGs page 2
3. Estimating exposure components of the regression models page 3
4. Further details on the data page 4
5. Further model outputs page 5
6. Sensitivity analysis page 7

1. Data sources

Table S1 provides details of the different data sources used in the study.

Table S1: Sources of data and information used in the study

Data	Source	Details
Mortality within 60 days of first laboratory-confirmed case or with confirmed COVID-19 present on death certificate	Public Health England (Now UK Health Security Agency)	By age band, CCG, week
New cases of laboratory-confirmed COVID-19	Public Health England	By age band, CCG, week
People onboarded to CO@h	NHS Digital: bespoke data collection from the programme aggregated by Imperial College London	By age band, CCG and fortnight, rounded to the nearest five patients or labelled as between one and seven.
Hospital admissions for COVID-19 or suspected COVID-19	Hospital Episode Statistics (HES)	Individual patient-level data aggregated by age band, fortnight and CCG of responsibility
In-hospital mortality	HES	Individual patient-level data
Lengths of hospital stay	HES	Individual patient-level data
Patient characteristics on admission	HES	Individual patient-level data
The proportion of acute beds occupied patients with Covid-19	NHS England and NHS Improvement	By acute trust, daily
The presence of a post-discharge COVID virtual ward	Kent, Surrey and Sussex Academic Health Sciences Network	By acute trust

2. Judging completeness of data within CCGs

We combined two sources of information to judge completeness of data:

(i) The management information collected by NHS Digital from each site;
(ii) Onboarding data received by the programme; and
(iii) Replies to the costing survey administered by the study team and sent to 28 sites.

The management information provided assessments as to whether the data reported by each site was complete up to mid-April 2021, the onboarding data covered the period from October 2020 to the end of April 2021 and the survey asked for numbers of individuals onboarded from the date the service started up to the end of April 2021.

For the 28 sites included in the survey, we compared the total numbers of onboarded individuals in the data we received from the programme (the programme data) to the numbers reported in the survey.

For most sites the numbers were broadly similar. However, among the CCGs reported as complete in the management information we excluded three CCGs where the numbers onboarded in the programme data were below 60% of those in the survey. We also included three CCGs where the data was not reported as complete but the numbers recorded as onboarded within the programme data were approximately the same as, or exceeded the numbers in the survey.

3. Estimating exposure components of the regression models

For our modelling of mortality and hospital admission we required estimates of exposure to COVID-19 so that we could then relate rates of outcome to levels of coverage and other variables. For example, for mortality, the basic regression model used is:

$$Log(number\ of\ deaths(t)) = Log(Exposure(t)) + \beta_0 + \beta_1(Coverage(t)) + \beta_2(Age\ band) + \beta_3(Month)$$

Where the β_i's are regression coefficients and *t* denotes the fortnight.

A simple approach would be to estimate exposure as the number of new cases in the same period as the deaths occurred, but, given many of those dying would have been identified as new cases some weeks before, this is unrealistic and would overestimate the exposure while cases are rising and underestimate it when cases are falling. A better approach would be to recognise the median time between diagnosis and death as about two weeks, and so use the number of new cases in the previous fortnight. In our study we went a further step and implemented an approach that applied weights to the case data from more than one previous time period. These weights reflect the relative contributions of each time period, sum to one, and can be estimated by linear regression, assuming the relationship remains constant over the period of the analysis (see Figure S1).

Figure S1: The application of weights to the current and previous time periods (fortnights) to create the exposure associated with outcomes. The sum of weights: $w_0 + w_1 + w_2 = 1$

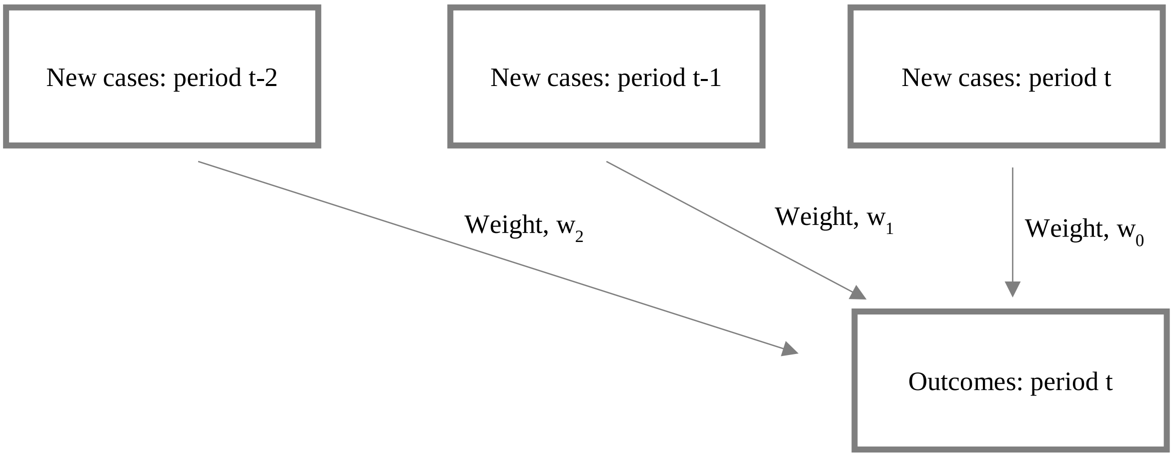

Assuming onboarding into the CO@h programme occurs soon after diagnosis, the lags and corresponding weights used for the onboarding data remain the same. The weighted onboarding numbers divided by the weighted new cases then becomes the coverage value that is used in the final regression model shown above.

The weights that we used are shown in Table S2. If we included lags of more than two fortnights, the estimated weights for those periods became very small and lacked statistical significance, so we carried out our final estimates by only going back as far as two previous fortnights. Different weightings were selected for the sensitivity analysis to see how they affected results.

Table S2: Weights applied to lagged numbers of new cases for each outcome. (w_0 is applied to new cases in the same period as the outcome is measured, w_1 is applied to new cases in the previous fortnight and w_2 to the fortnight before that.)

		**Weight**		
**Outcome**	**Age band**	w_0	w_1	w_2
Mortality	65 to 79	23.1%	60.2%	16.6%
	80+	27.5%	67.4%	5.0%
Hospital admission	65 to 79	61.1%	37.8%	1.1%
	80+	81.8%	14.5%	3.7%

.

4. Further details on the data

Time series plots of numbers of newly diagnosed cases and numbers onboarded to the programme within the selected 37 CCGs over the study period (2nd November 2020 and 21 February 2021) are shown in Figure S2. New diagnoses peaked in the fortnight straddling the new year, whilst onboarding peaked in the following fortnight.

Figure S2 a: Numbers of new diagnoses of COVID-19 by fortnight. b: Numbers of people onboarded to the CO@h programme. Data for the 37 CCGs included within the analysis.

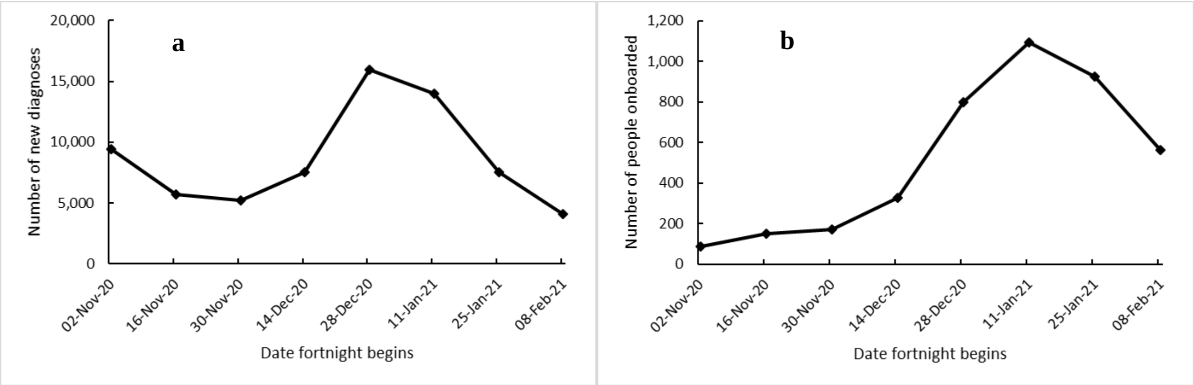

Profiles of all COVID-19 deaths and admissions for confirmed or suspected COVID-19 are shown in Figure S3.

Figure S3: Outcomes by fortnight within the 37 CCGs. a: All deaths from COVID-19, b: Hospital admissions for confirmed or suspected COVID-19

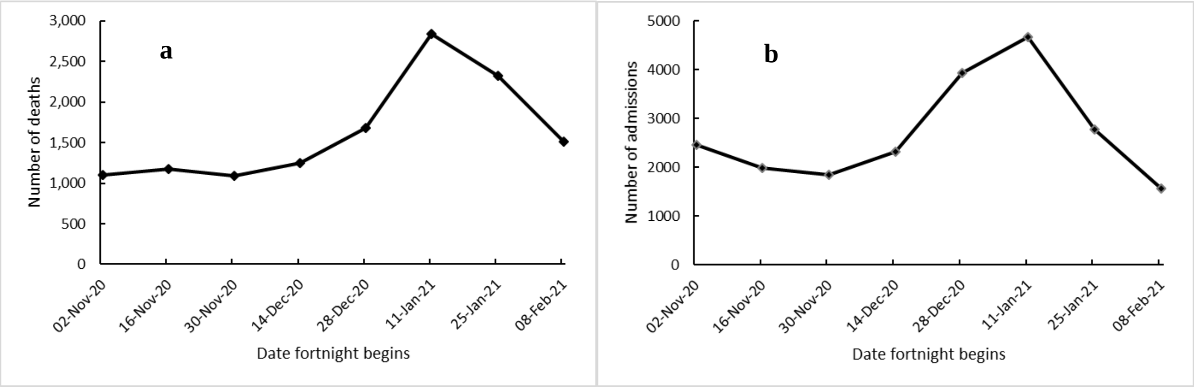

Rates of in-hospital mortality and median lengths of stay for patients admitted to hospital with confirmed or suspected COVID-19 are shown in Figure S4. Mortality rates peak at approximately 30% over the time of the

new year and decline thereafter to around 21%. Median lengths of stay decline from the end of November while admission numbers increase.

Figure S4: In-hospital outcomes by fortnight for patients resident within the 37 CCGs and admitted with confirmed or suspected COVID-19. a: In-hospital mortality, b: Median length of stay.

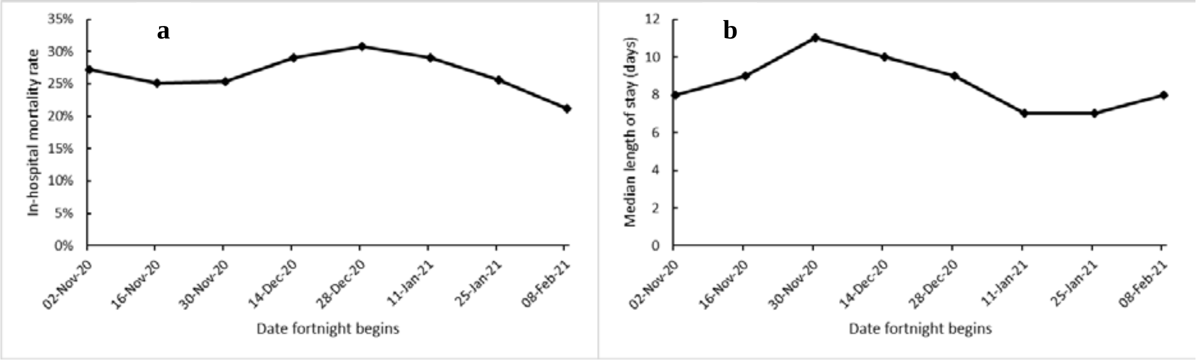

5. Further model outputs

Figures S5 to S8 illustrate the impact each variable has on each outcome. The actual values are presented in the main paper. In each case the 65 to 79 age band and the month of November are reference categories against which the other age bands and months are compared.

Figure S5: Impact of each variable used in the multivariate model on the risk of death (mortality rate ratios). Error bars denote 95% confidence intervals.

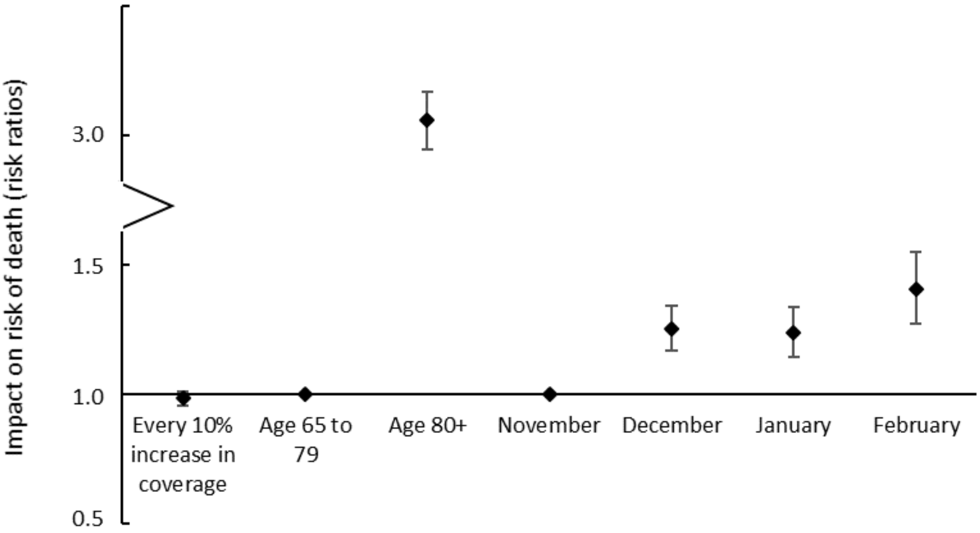

Figure S6: Impact of each variable used in the multivariate model on the risk of hospital admission (admission rate ratios). Error bars denote 95% confidence intervals.

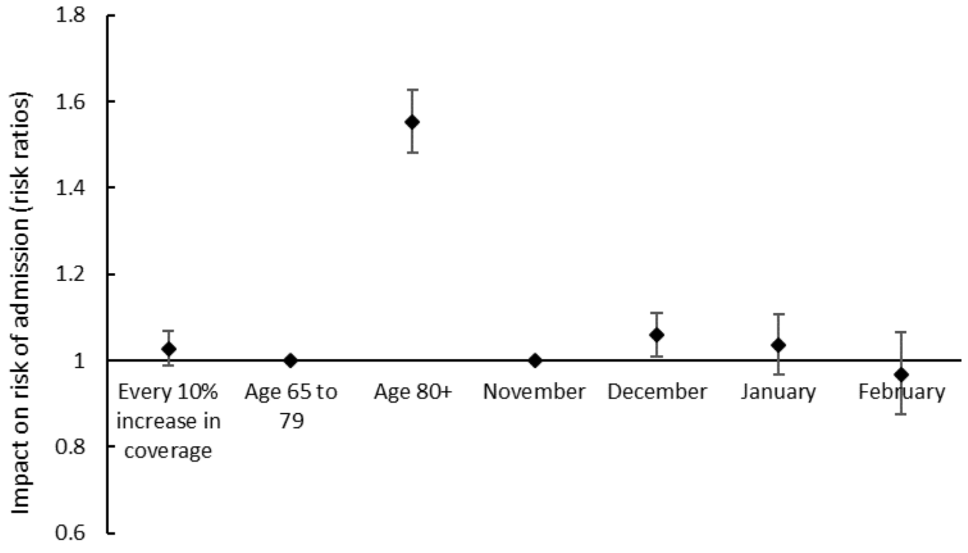

Figure S7: Impact of each variable used in the multivariate model on the risk of in-hospital mortality for people admitted for confirmed or suspected COVID-19 (odds ratios). Error bars denote 95% confidence intervals.

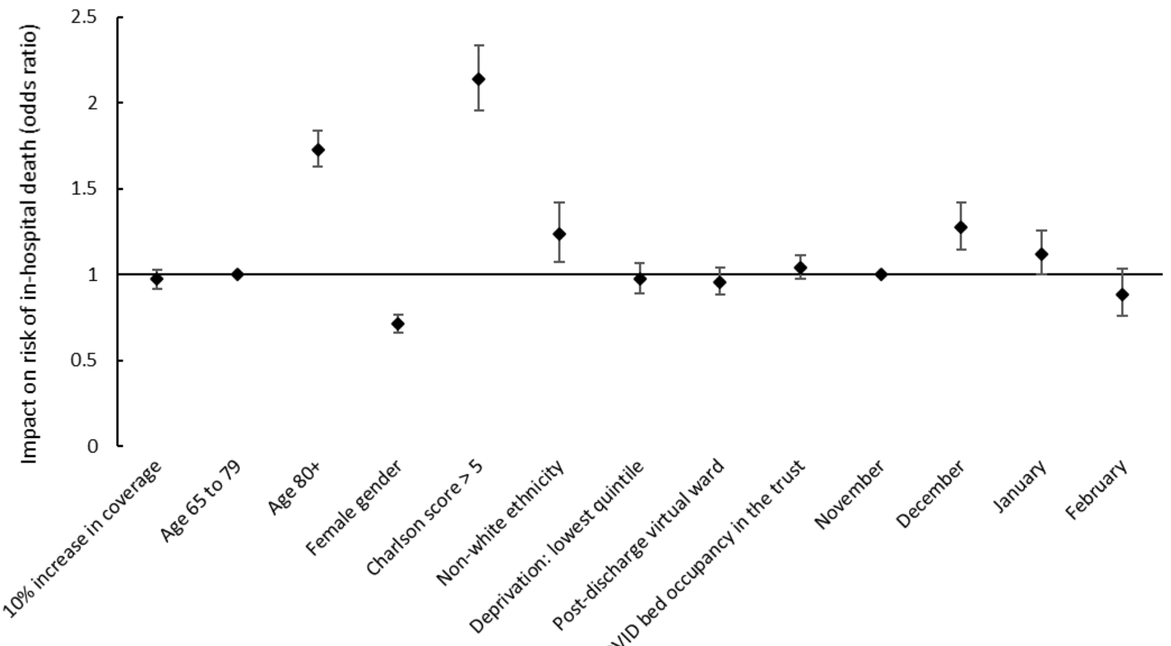

Figure S8: Impact of each variable used in the multivariate model on length of stay for people admitted for confirmed or suspected COVID-19 (% change). Error bars denote 95% confidence intervals.

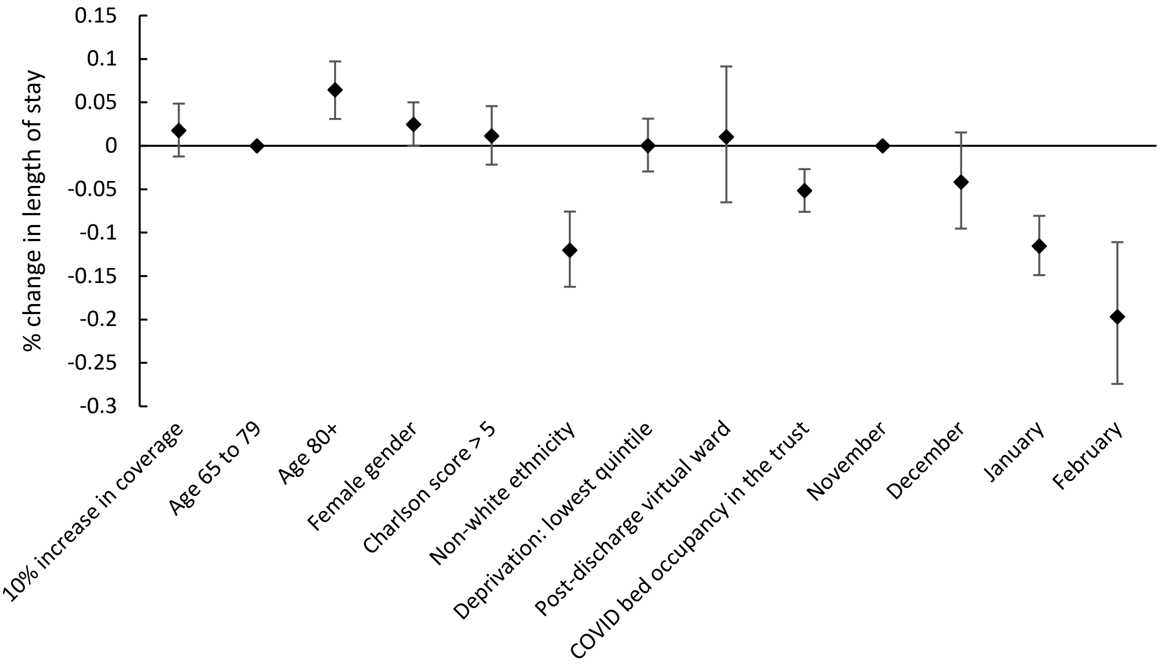

The correlations between repeated measures for each multivariate model where we used General Estimating Equations, are shown in Table S3.

Table S3: Exchangeable working correlations between repeated measures from each multivariate model

Model		Correlation
Main outcome models	Mortality from COVID-19	0.113
	Hospital admission	0.214
	In-hospital mortality	0.003
	Length of stay	0.012
Models of patient characteristics on admission	Gender	0.009
	Charlson score	0.079
	Ethnicity	0.816
	Deprivation	0.782
	Age	0.297

6. Sensitivity analysis

For sensitivity analysis we tested different scenarios for weighting lagged variables to create different values for exposure in our regression models. We also investigated outcomes if we excluded hospital admissions for suspected COVID-19, focussing exclusively on confirmed diagnoses. For the weighting scenarios we chose the same weighting for both age bands and varied them across a range of feasible values. For the in-hospital outcomes the weightings are applied to the coverage and correspond to those for admissions.

Under each scenario, the impacts of a 10% increase in coverage on each outcome are shown in Tables S4 to S6. None of the effects are statistically significant at the 5% level (two-sided), although the impact on the risk of hospital admission without any lags (w_0 = 100%, w_1 = 0%, w_2 = 0%), or with a lag of just one fortnight (w_0 = 0%, w_1 = 100%, w_2 = 0%) are borderline significant for a positive relationship (p=0.06 in both scenarios).

Table S4: The impact of coverage on the risk of mortality under different modelling assumptions

Scenario		Relative risk of death associated with a 10% increase in coverage *(95% confidence interval)*	
Baseline	(see table 2)	0.98	*(0.96, 1.01)*
Weighting (applied to both age bands)	w_0 = 30%, w_1 = 50%, w_2 = 20%	0.98	*(0.95, 1.01)*
	w_0 = 10%, w_1 = 70%, w_2 = 20%	0.98	*(0.95, 1.01)*
	w_0 = 30%, w_1 = 70%, w_2 = 0%	1.00	*(0.97, 1.03)*
	w_0 = 0%, w_1 = 100%, w_2 = 0%	1.00	*(0.97, 1.02)*

.

Table S5: The impact of coverage on the occurrence of hospital admission under different modelling assumptions

Scenario		Relative risk of admission associated with a 10% increase in coverage *(95% confidence interval)*	
Baseline	(see table 2)	1.03	*(0.99, 1.07)*
Weighting (applied to both age bands)	w_0 = 60%, w_1 = 40%, w_2 = 0%	1.02	*(0.98, 1.06)*
	w_0 = 100%, w_1 = 0%, w_2 = 0%	1.03	*(1.00, 1.07)*
	w_0 = 0%, w_1 = 100%, w_2 = 0%	1.05	*(1.00, 1.10)*
	w_0 = 50%, w_1 = 50%, w_2 = 0%	1.02	*(0.98, 1.06)*
	w_0 = 60%, w_1 = 30%, w_2 = 10%	1.01	*(0.97, 1.05)*
Exclude patients with suspected COVID-19 as primary diagnosis		1.01	*(0.97, 1.04)*

.

Table S6: The impact of coverage on in-hospital mortality and length of stay under different modelling assumptions

Scenario	Odds ratio associated with in-hospital mortality for every 10% increase in coverage *(95% confidence interval)*		Relative change in length of stay for every 10% increase in coverage *(95% confidence interval)*	
Baseline	0.97	*(0.92, 1.03)*	1.8%	*(-1.2%, 4.9%)*
Weighting used to create coverage variable (applied to both age bands)				
w_0 = 60%, w_1 = 40%, w_2 = 0%	0.96	*(0.91, 1.02)*	1.7%	*(-1.4%, 4.9%)*
w_0 = 100%, w_1 = 0%, w_2 = 0%	0.98	*(0.93, 1.02)*	1.2%	*(-1.3%, 3.7%)*
w_0 = 0%, w_1 = 100%, w_2 = 0%	0.95	*(0.90, 1.01)*	0.9%	*(-1.7%, 3.6%)*
w_0 = 50%, w_1 = 50%, w_2 = 0%	0.96	*(0.90, 1.02)*	1.7%	*(-1.4%, 5.0%)*
w_0 = 60%, w_1 = 30%, w_2 = 10%	0.96	*(0.91, 1.02)*	2.1%	*(-1.1%, 5.4%)*
Exclude patients with suspected COVID-19 as primary diagnosis	0.98	*(0.92, 1.04)*	0.2%	*(-2.8%, 3.3%)*
